# Structural-Dynamics and Binding Analysis of RBD from SARS-CoV-2 Variants of Concern (VOCs) and GRP78 Receptor Revealed Basis for Higher Infectivity

**DOI:** 10.3390/microorganisms9112331

**Published:** 2021-11-11

**Authors:** Abbas Khan, Anwar Mohammad, Inamul Haq, Mohammad Nasar, Waqar Ahmad, Qudsia Yousafi, Muhammad Suleman, Sajjad Ahmad, Aqel Albutti, Taimoor Khan, Sulaiman K. Marafie, Eman Alshawaf, Syed Shujait Ali, Jehad Abubaker, Dong-Qing Wei

**Affiliations:** 1 Department of Bioinformatics and Biological Statistics, School of Life Sciences and Biotechnology, Shanghai Jiao Tong University, Shanghai 200240, China; abbaskhan@sjtu.edu.cn (A.K.); taimor.khaan@sjtu.edu.cn (T.K.); 2 Department of Biochemistry and Molecular Biology, Dasman Diabetes Institute, Dasman 15462, Kuwait; anwar.mohammad@dasmaninstitute.org (A.M.); sulaiman.marafie@dasmaninstitute.org (S.K.M.); eman.alshawaf@dasmaninstitute.org (E.A.); jehad.abubakr@dasmaninstitute.org (J.A.); 3 Department of Animal Sciences, Jeonbuk National University, 567 Baekji-Daero, Deokjin-gu, Jeonju-si, Jeollabuk-do, Jeonju 54896, Korea; inam7353@gmail.com; 4 Animal Biotechnology Division, National Institute of Animal Science, Rural Development Administration, Wanju-gun 55365, Korea; 5 Department of Biosciences, COMSATS University, Islamabad-Sahiwal Campus, Punjab 57000, Pakistan; mnasar325@gmail.com (M.N.); qudsia@cuisahiwal.edu.pk (Q.Y.); 6 Department of Microbiology, Abdul Wali Khan University (AWKUM), Mardan 23200, Khyber Pakhtunkhwa, Pakistan; waqarahmad7120@gmail.com; 7 Center for Biotechnology and Microbiology, University of Swat, Swat 19200, KP, Pakistan; suleman@uswat.edu.pk (M.S.); shujaitswati@uswat.edu.pk (S.S.A.); 8 Department of Health and Biological Sciences, Abasyn University, Peshawar 25120, Pakistan; sahmad@bs.qau.edu.pk; 9 Department of Medical Biotechnology, College of Applied Medical Sciences, Qassim University, Buraydah 51452, Saudi Arabia; as.albutti@qu.edu.sa; 10 State Key Laboratory of Microbial Metabolism, Shanghai-Islamabad-Belgrade Joint Innovation Center on Antibacterial Resistances, Joint Laboratory of International Cooperation in Metabolic and Developmental Sciences, Ministry of Education and School of Life Sciences and Biotechnology, Shanghai Jiao Tong University, Shanghai 200030, China; 11 Peng Cheng Laboratory, Vanke Cloud City Phase I Building 8, Xili Street, Nashan District, Shenzhen 518055, China

**Keywords:** COVID-19, SARS-CoV-2, new variants, GRP78, infectivity

## Abstract

Glucose-regulated protein 78 (GRP78) might be a receptor for SARS-CoV-2 to bind and enter the host cell. Recently reported mutations in the spike glycoprotein unique to the receptor-binding domain (RBD) of different variants might increase the binding and pathogenesis. However, it is still not known how these mutations affect the binding of RBD to GRP78. The current study provides a structural basis for the binding of GRP78 to the different variants, i.e., B.1.1.7, B.1.351, B.1.617, and P.1 (spike RBD), of SARS-CoV-2 using a biomolecular simulation approach. Docking results showed that the new variants bound stronger than the wild-type, which was further confirmed through the free energy calculation results. All-atom simulation confirmed structural stability, which was consistent with previous results by following the global stability trend. We concluded that the increased binding affinity of the B.1.1.7, B.1.351, and P.1 variants was due to a variation in the bonding network that helped the virus induce a higher infectivity and disease severity. Consequently, we reported that the aforementioned new variants use GRP78 as an alternate receptor to enhance their seriousness.

## 1. Introduction

Coronaviruses have posed severe health concerns to the world in the 21st century, reflected by their long-term persistence, enhanced ability of invasion, and the emergence of novel variants of SARS-CoV-2. Specifically, the interactions of the host ACE2 (angiotensin-converting enzyme) receptor with the spike protein are critical for viral propagation and pathogenesis [1]. Therefore, the virus’ spike protein is a significant target for designing antiviral strategies [2,3]. The two main subunits, S1 and S2, of the RBD facilitate spike protein–host receptor interactions, leading to viral entry and transmission [4]. In contrast, recent investigations also depict novel interaction patterns involving a 78-kilodalton glucose-regulated protein (GRP78) and the RBD, suggesting alternative entry mechanisms for SARS-CoV-2 [5]. GRP78, also known as BiP, is a main HSP70 family member encoded by the HSPA5 gene, where it resides in the endoplasmic reticulum (ER) and plays a key role in protein folding [4,5]

Additionally, GRP78 also serves as a significant regulator involved in the unfolded protein response and mediates cellular adaptation under ER-specific stress [6]. GRP78 has a higher expression level compared to ACE2 in several tissues, such as the epithelial cells of the bronchi and the mucosa of the respiratory tract [7]. Similarly, serum-based higher levels of GRP78 are also common in SARS-CoV-2-mediated respiratory diseases [7]. Studies have reported that severe viral infections promote cellular translocation of GRP78 and alter its function as a co-receptor to mediate cellular viral entry processes [8]. The bat coronaviruses HKU9 and MERS-CoV spike proteins are also known to interact with GRP78 to facilitate cellular attachment and viral entry [9,10]. Viral infection is enhanced through induced ER stress conditions and higher expression levels of total and cell surface GRP78 (csGRP78) receptors [7]. GRP78-mediated cellular recognition and increased transmissibility in the B.1.1.7 and B.1.351 variants have also been found to be co-related with SARS-CoV-2 [11,12]. The GRP78 substrate-binding domain (SBD) is the binding site for the SARS-CoV-2 spike RBDs, particularly the region between C480–C488. The GRP78 substrate-binding domain (SBD) is the docking domain for the SARS-CoV-2 spike RBD’s C480–C488 region. The proposed host-cell receptor, ACE2, is likely to bind to the spike through the CS-GRP78 region. A new experimental analysis by Lee et al. demonstrated GRP78 in conjunction with both ACE2 and the spike proteins after SARS-CoV-2 infection of cells, corroborating the initial hypothesis. According to the findings, SBD is critical for binding [13]. Therefore, it is important to elucidate the interaction patterns of GRP78 and the SARS-CoV-2 variants. This may reveal the factors involved in mediating the viral entry process and help in the design of strategies for the management of the current pandemic.

The emerging variants of SARS-CoV-2 are alarming due to their more virulent properties, such as increased contagiousness, immune evasion, and severe disease outcomes. Variants of concern (VOCs), including B.1.1.7, B.1.351, B.1.617, B.1.618, R.1, and P.1 carry numerous RBD domain-specific mutations in the spike protein. These RBD-specific mutations include N501Y, E484K, L452R, and K417N/T [14,15,16]. Stronger binding of the RBD-ACE2 complex due to these mutations is also linked with higher infectivity of SARS-CoV-2 variants [13,17]. Several studies have focused on examining viral RBD-host ACE2 receptor interactions; however, other interacting receptors need to be further investigated.

It is vital to understand the proteome of SARS-CoV-2 to develop therapeutics-related and proteomics-based methods against COVID-19. Recently, researchers have used several approaches, including determining the evolutionary relationship, developing vaccines, identifying the mutational landscape, and developing novel therapeutics to better challenge SARS-CoV-2 associated health concerns [17]. These treatments include activating a boosted immune response to fight against pathogens or hindering pathogen-receptor interactions from blocking viral attachment and entry into the host cell [18]. Therefore, a comprehensive examination is required to determine the impact of these RBD-specific substitutions on spike protein bonding with GRP78 receptors and explore the associated structural and functional consequences. In the current work, several techniques, including protein–protein docking and biophysical approaches, were used to analyze the structural modifications that alter RBD-GRP78 receptor binding and its potential impact on infectivity. The study provides mechanistic insights to explore the RBD further and specific structural changes to aid future SARS-CoV-2 research.

## 2. Materials and Methods

### 2.1. Variants Modeling and Preparation

Recently reported variants of SARS-CoV-2, i.e., B.1.1.7, B.1.351, B.1.617, and P.1, which spread exponentially and pose a serious threat to human health, were modeled. The sequence of SARS-CoV-2 spike protein (accession number: P0DTC2) RBD were retrieved from the UniProt database, and mutations were introduced [18]. Mutations were introduced in B.1.1.7, variant N501Y; B.1.351, variants K417N, E484K, and N501Y; B.1.617 (L452R); and in P.1, variants K417T, E484K, and N501Y. Modeler *version 14.0* was used to execute the homology modeling of variant sequences. Due to sequence similarity using PSI-BLAST, the experimentally reported structures (6XDG (E) and 6M0J (E) were reported to be the best matches and were used as templates for variant modeling. The modeled structures were prepared and minimized using Chimera’s steepest gradient minimization algorithm [19,20]. The modeled structures were validated using the Ramachandran plot and ERRAT. For structural comparison, the wild-type structure with accession number: 6M0J was retrieved from RCSB [19,21], and the variants were compared. The crystal structure of GRP78 is available in the RCSB, which was retrieved by using accession number 6ASY [21].

### 2.2. Docking of Variants with the Host GRP78 Receptor

The high ambiguity-driven protein–protein docking (HADDOCK) server ( The Utrecht Biomolecular Interactions software portal) was used to execute the variant docking with the GRP78 receptor using the standard protocol [22]. This biochemical and/or biophysical interaction data analysis method uses chemical shift perturbations information from NMR titration assays or mutagenesis data. To complete the docking process, this data is supplied as Ambiguous Interaction Restraints (AIRs). Ambiguous proximity among all residues proven to be engaged in the interaction is described as an AIR. For this purpose, the rigid body docking conformations were kept at 1000, while during the refinement, the flexible docking conformations were kept at 200 using the solvated docking option to understand the impact of interfacial water molecules. The best cluster was then retrieved for further analysis.

### 2.3. Estimation of Dissociation Constant (K_D_)

The strength of macromolecular complexes, particularly protein–protein or antibody, can be demonstrated by calculating the K_D_ kinetics. Determination of the dissociation constant provides a better view of biological systems, their role in diseases, and therapeutic development. However, the protein binding energy prediction (PRODIGY) server was used for the calculation of the dissociation constant (K_D_) [23]. This server uses a highly accurate predictive model by considering the interface contacts and features of non-interface residues to estimate the interacting partners’ binding strength.

### 2.4. Dynamics Features of Wild-Type RBD and Variants-GRP78 Complexes

In our study, we used the strategy of a molecular dynamics simulation run at 300 ns to observe the structural dynamics of the wild-type RBD-GRP78 and B.1.1.7-RBD-GRP78, B.1.351-RBD-GRP78, and P.1-RBD-GRP78 complexes. Using the AMBER20 simulation package employing *FF19SB*, structural coordinates and topologies were generated [24,25]. The standard method used by Abbas et al., 2021 was followed for the system preparation and running of MD simulation [14]. In summary, the systems were neutralized by adding the proper number of counterions and solvated in a TIP3P solvation box. System minimization was achieved via 6000 and 3000 steps of steepest descent and conjugate gradient algorithms. The heating of the systems was done to 300 °K, followed by the equilibration phase. The equilibrium for each system was attained at almost 50 ns, and then the final stage production runs for each system was run at a time scale of 400 ns. The trajectories of the simulation were generated and analyzed through the CPPTRAJ and PTRAJ modules of AMBER [26].

### 2.5. Estimation of Binding Free Energy

The AMBER MMGBSA.py module was used for the calculation of binding free energies for both the wild-type and variant complexes [27]. The electrostatic, van der Waals, polar, and non-polar energies were analyzed by subjecting the system to the MM/GBSA method [28]. Furthermore, the system’s net binding free energy was also estimated using the following equation:

(1) $$\Delta G_{net\;binding\;energy}=\Delta G_{complex\;binding\;energy}-\left[\;\Delta G_{receptor\;binding\;energy}+\Delta G_{ligand\;binding\;energy}\right]$$

Each of the above components of net binding energy can be split as follows:

(2) $$G=G_{bonded}+G_{van\;der\;waals}+G_{polar\;solvation\;energy}+G_{non-polar\;solvation\;energy}$$

The entropic calculation was not conducted since it is a computationally expensive and time-consuming process and is also highly susceptible to significant inaccuracies [29].

## 3. Results and Discussion

### 3.1. Structural Modeling and Analysis

**Figure 1:**
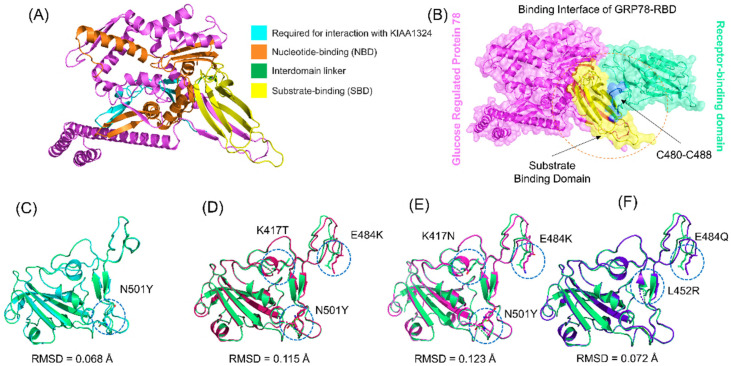
Structural modeling and analysis of GRP78 and spike RBD of the wild-type and docking interaction analysis B.1.1.7, P.1, B.1.351, and B.1.617 variants. (**A**) shows the structure of GRP78 and its domain organization; (**B**) shows the binding interface of GRP78 and spike RBD; (**C**) shows the superimposed structure of wild-type and B.1.1.7 variant RBD; (**D**) shows the superimposed structure of wild-type and P.1 variant RBD; (**E**) shows the superimposed structure of wild-type and B.1.1.7 variant RBD; (**F**) shows the superimposed structure of wild-type and B.1.617 variant RBD.

The emergence of SARS-CoV-2 and its variants around the world has caused a serious threat to human health and safety. In late December 2019, the emergence of the ongoing pandemic caused by a novel coronavirus (SARS-CoV-2) was announced. It was reported that this virus enters the host cell through spike RBD and ACE2 fusion, and this interaction is critical for viral propagation and pathogenesis [1]. The spike protein of the virus is thus a significant target in the design of antiviral strategies. The two main subunits (S1 and S2) of the RBD facilitate spike protein–host receptor interactions, leading to viral entry and transmission [4]. The primary investigation reported that the spike interacts with ACE2 of the host cell, but later it was discovered that this virus might also use alternate receptors, such as GRP78 and neuropilin-1 [11,30]. This was reported in recent investigation, that GRP78 mediates alternate entry mechanism for SARS-CoV-2 [12]. Similarly, GRP78 mediated enhanced cellular recognition in the B.1.1.7 variant, and higher transmissibility of the B.1.351 variant has also been co-related with SARS-CoV-2 [11,12]. Understanding the role of GRP78 is only the tip of the iceberg. The emergence of new variants of SARS-CoV-2 has created an alarming situation due to properties that include higher contagiousness, immune evasion, and severe disease outcomes. These different VOCs, B.1.1.7, B.1.351, B.1618, R.1, B.1.617, and P.1, carry numerous RBD domain-specific mutations on the spike protein, including N501Y, E484K, L452R, and K417N/T [12,14,15]. Stronger binding of the RBD-ACE2 complex due to these mutations is also linked with higher infectivity of SARS-CoV-2 variants [13,17]. Several studies have focused on examining viral RBD-host ACE2 receptor interactions, but other interacting receptors need further investigation. To understand how these variants affect the binding of RBD and GRP78, the current study explored the atomic-level features of the interactions and structural dynamics properties. The structure of GRP78 was retrieved from RCSB. GRP78 contains NBD, SBD, and an interdomain linker (Figure 1A). Structural modeling of RBD from B.1.1.7, B.1.351, B.1.617, and P.1 variants was carried out by introducing the variant-specific mutations and comparing them with the wild-type. A previous study reported that residues C480–C488 are the binding residues for SARS-CoV-2 RBD to directly interact with the SBD of GRP78. The binding interface of GRP78 and RBD is shown in Figure 1B. Comparative structural analysis of the wild-type and variants revealed changes in the structures and RMSD. The RMSD difference between wild-type and B.1.1.7 was 0.068 Å; for P.1, the RMSD difference was 0.115 Å; for B.1.351, the RMSD difference was 0.123 Å; and for B.1.617, the RMSD difference was 0.072 Å. The superimposed structures of the wild-type RBD and the variants are given in Figure 1C–F. This shows that the mutations have caused structural variation and thus need further investigation to reveal the specific features that may increase the binding and alter the dynamics.

### 3.2. Docking of Spike RBDs and GRP78

**Figure 2:**
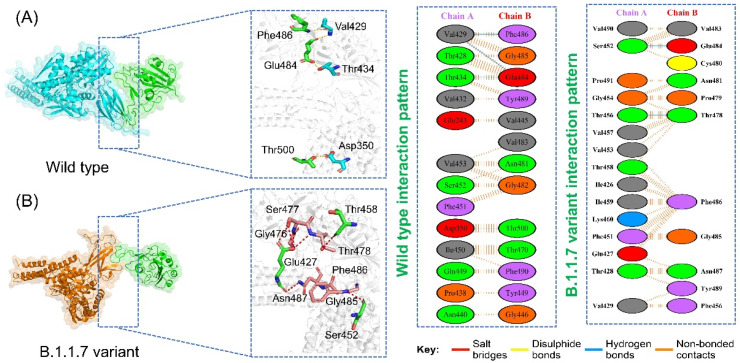
Binding modes of GRP78 and spike RBD of the wild-type and B.1.1.7 variant. (**A**) shows the interaction of GRP78 and RBD of the wild-type, while (**B**) shows the interaction of GRP78 with B.1.1.7-RBD. The right panels show the 2D interaction patterns of the wild-type and B.1.1.7.

Protein interactions and networks are crucial for regulating cellular processes and activities. Investigation of protein interactions by determining their strength plays a critical role in understanding the biological relevance of the macromolecular association. Any discrepancy in protein–protein interrelationships can result in physiologically defective pathways, which can lead to pathological conditions. The interaction of proteins in cells is a complicated process; in many illnesses, the interacting interface is a significant target for clinical application. Any mutation at the interface site can influence binding and activity directly. Exploring the binding of various proteins, specifically SARS-CoV-2, would help in the development of novel treatment methods. This may also unveil the interactions of RBD and spike glycoprotein with the host receptors involved in progression of the infection. In this regard, docking of RBD and ACE2 has been reported previously as an effective strategy to understand how SARS-CoV-2 interacts with the host and triggers the infection process. Here, a similar approach was employed using the HADDOCK docking algorithm to determine the binding mode of the GRP78 with the wild-type and variant RBDs. From the docking analysis, it was interpreted that most of the interacting residues are the same; however, some difference between the wild-type and variant interacting residues was observed. In the case of the wild-type, the docking score was reported to be −205.36 kcal/mol with four hydrogen bonds and 67 non-bonded contacts. As shown in Figure 2A, the wild-type spike with the GRP78 receptor formed four hydrogen bonds by interacting with residues Glu484-Thr434, Phe486-Val429, and Thr500-Asp350. Glu484 has been previously reported to be actively involved in interaction with GRP78 [11]. Similarly, a total of seven hydrogen bonds and two salt bridges were formed, and the docking score for B.1.1.7 was reported to be −289.67 kcal/mol (Figure 2B). The interacting residues of the B.1.1.7 variant comprise Ser452, Glu427, Gly476, Ser477, Thr458, Thr478, Gly485, Phe486, and Asn487. The interaction formed by Asn487 and Ser452 are also preserved here and are reported to be critical for stronger interactions and recognition [11,14].

**Figure 3:**
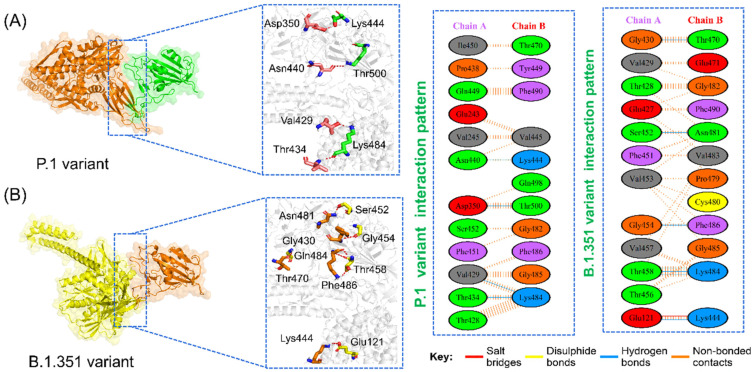
Binding modes of GRP78 and spike RBD of P.1 and B.1.351 variants. (**A**) shows the interaction of GRP78 and RBD of P.1, while (**B**) shows the interaction of GRP78 with B.1.351-RBD. The right panels show the 2D interaction patterns of P.1 and B.1.351.

Furthermore, the interaction pattern of the P.1 variant was also explored, and it revealed a similar pattern of interactions as the wild-type by making contacts between Asp350, Asn440, Lys444, Val429, Thr434, Lys484, and Thr500 (Figure 3A). The docking score for the P.1 variant was reported to be −279.59 kcal/mol. It is important to note that the mutated residue Lys484 formed two interactions with the key residues Val429 and Thr434 of GRP78, which could act as a potential distinct feature of infection. This residue was also reported to form two additional bonds in the case of the ACE2 receptor [14,15]. This means the binding residues of the wild-type and the P.1 variant are almost the same; however, the latter tends to show greater residue preference than the former.

The B.1.351 variant favors binding with Glu121, Gly430, Lys44, Ser452, Asn481, Gly454, Thr458, Gln484, and Phe486. It is worth mentioning that the mutated residue Lys484 in B.1.351 forms three hydrogen bonds and is the only salt bridge between Lys444 and Glu121. The total binding energy for this variant is −265.66 kcal/mol with 7 hydrogen bonds, 1 salt bridge, and 70 non-bonded contacts. The interaction pattern of the B.1.351 variant is given in Figure 3B.

**Figure 4:**
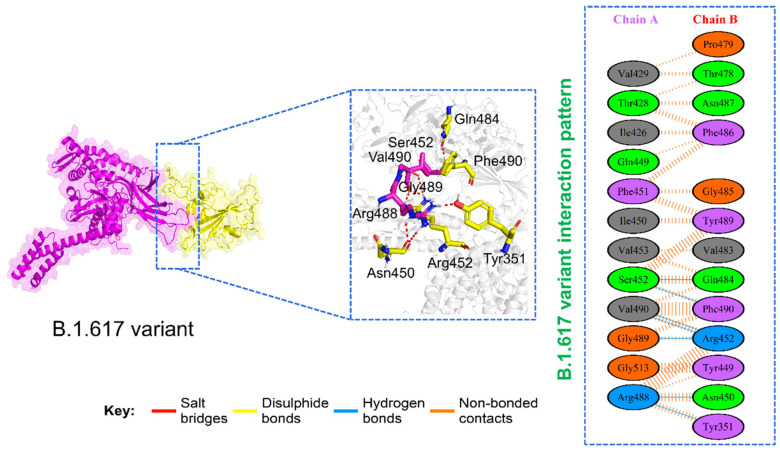
Binding modes of GRP78 and spike RBD of the B.1.617 variant. The figure shows the interaction of GRP78 and RBD of B.1.617, while the 2D interaction pattern of B.1.617 is displayed on the right panel.

On the other hand, the interacting network of the B.1.617 variant includes Tyr351, Asn450, Arg452, Arg488, Gly489, Val490, Ser451, Gln484, and Phe490 residues. With a total of seven hydrogen bonds, the docking score was reported to be −226.32 kcal/mol. The bonding network of the B.1.617 variant is given in Figure 4. Our findings are consistent with previous experimental reports, as GRP78 mediated enhanced cellular recognition in the B.1.1.7 variant, and the higher transmissibility of the B.1.351 variant has also been co-related with SARS-CoV-2 [11,12]. This reveals that the reported variants may also use GRP78 for enhanced transmission and pathogenesis. Moreover, the increased number of hydrogen bonds and salt bridges by the B.1.1.7, B.1.351, and P.1 variants is firmly in uniformity with previous docking and simulation studies on GRP78 and ACE2 [12]. The docking scores and K_D_ calculations are given in Table 1.

### 3.3. Conformational Dynamics Analysis by RMSD

**Figure 5:**
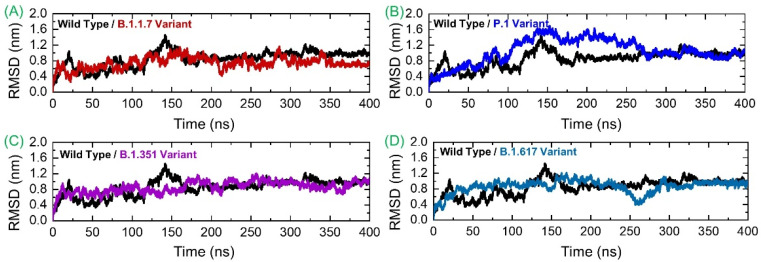
Structural and dynamic stability of GRP78 and spike RBD of the wild-type and variants calculated as RMSD. (**A**) shows the RMSD of wild-typeRBD-GRP78 complex; (**B**) shows the RMSD of B.1.1.7-RBD-GRP78 complex; (**C**) shows the RMSD of P.1-RBD-GRP78 complex; (**D**) shows the RMSD of B.1.351-RBD-GRP78 complex.

Deciphering conformational dynamics of the SARS-CoV-2 spike variants/wild-type complexed with the human GRP78 receptor is key to a better explanation of the complex’s stability/instability and prediction of the mutation’s impact on the structure, function, and overall binding. This further implicates the virus fitness for the host in terms of attachment, infection, and transmissibility. Conformational dynamics of the analyzed complexes were understood via calculating RMSD with respect to the 400 ns time period. As shown in Figure 5A, the wild spike-GRP78 complex is subject to continuous fluctuations until 185 ns. It gained equilibrium and showed dynamics with a net RMSD of ~0.8 nm until the end of the simulated time. The highest RMSD surge reaches ~1.4 nm at 140 ns. It seems that the interaction between these two molecules was initially unstable and experienced several structural adjustments to obtain the fittest conformation with respect to each other. The structural adjustments allow the intermolecular binding to become highly stable with time, and the stable binding mode of the molecules could be achieved at the end. The GRP78-B.1.1.7 RMSD is more stable compared to the wild-type, although the plot is inconsistent. The maximum RMSD reported for this system reached 0.9 nm, while the lowest RMSD revealed was around 0.3 nm. Thus, it can be inferred that the GRP78-B.1.1.7 interaction experienced fewer structural variations in which few bonds were broken, and some new bonds were established to strengthen the binding. The structure showed more stable behavior between 350–400 ns. The GRP78-P.1 variant depicted stability until 50 ns with deviation around 0.4 nm and then experienced a minor structural deviation phase until 90 ns. Afterward, the system RMSD continuously upsurged, reaching an RMSD of 1.6 nm. RMSD was observed to be in continuous decline toward the end, achieving a mean of approximately 0.8 nm. During the last 150 ns (250–400) the structure gained significant stability and ended up with 0.8 nm. The GRP78-B.1.351 variant RMSD’s behavior was stable until 210 ns, followed by a minor variability phase, and then gained significant stability at the end. Similarly, this complex also demonstrated greater stability during the last 150 ns. The GRP78-B.1.617 variant exhibited stability for the first 150 ns, with an RMSD of ~0.6 nm, then experienced a small surge and slowly declined until 260 ns, followed by a sudden increase at the end, where RMSD reached 0.8 nm and remained consistent until the end of the simulation. These findings suggest a stable evolution of these variants and thus support the stable binding pattern. Therefore, our inferences are in strong correlation with the findings of previous studies. As previously reported, global RBD stability contributes to ACE2-binding affinity [31]. A strong relationship between the RBD stability and affinity is corroborated by previous findings, in which mutations that increase the structural stability and rigidity escort upsurges in binding affinity [32,33]. For instance, other findings, which reported a destabilizing mutation C432D in RBD, lessened ACE2 assisted entry to the cell using a spike trimer [31]. In the recently reported mutations, those in UK, South Africa, Brazil and other countries, the stability also increased and claimed a stable evolution of the new variants. Studies have shown clearly that the environmental pH plays a key part in SARS-CoV-2 infection as demonstrated by the pH (6.3) requirement for virus entry and its genome release (pH < 6) endosomes along with pH of the export secretory pathway (pH 5.5) for newly assembled viruses [34,35]. pH variation with respect to SARS-CoV-2 variant cell entry and exit has been discussed. It is reported that the D398 variant allows pH-dependence open/close equilibrium of the spike, which is coupled potentially to the impact of D614G mutation and linoleic acid-binding. This is also possibly linked to A570D mutation [34]. Also, it is reported that pH does not trigger the conformational changes of the spike protein from the “down” to the “up” state [36]. As can be exemplified by no significant changes in the spike protein conformation in CryoEM at pH 5.6 in contrast to the neutral-pH for SARS-CoV-1 [37]. Previous studies have shown a close association between RBD stability and affinity, with mutations that maximize structural stability and inflexibility causing upsurges in binding affinity [14,15]. The RMSDs of all the complexes are given in Figure 5.

### 3.4. Investigating System Compactness

**Figure 6:**
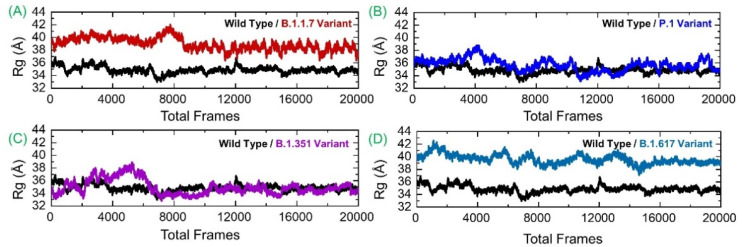
Structural compactness of GRP78 and spike RBD of the wild-type and variants calculated as Rg. (**A**) shows the Rg of wild-type-RBD-GRP78 complex; (**B**) shows the Rg of B.1.17-RBD-GRP78 complex; (**C**) shows the Rg of P.1-RBD-GRP78 complex; (**D**) shows the Rg of B.1.351-RBD-GRP78 complex.

Next, the compactness of systems was analyzed using the radius of gyration analysis. All the systems reported varying compactness. For example, the GRP78-B.1.1.7 variant was more compact for the first half of simulation time, then faced a minor surge and a decline, which remained consistent and in strong equilibrium toward the end. It can be seen that until 150 ns, the Rg value remained high relative to the last 150 ns. The Rg value during the initial 150 ns remained at 39.0 Å, which then decreased to 37.0 Å during the last 150 ns. In contrast, the GRP78-wild-type, as demonstrated in RMSD analysis, achieved stability in short phases but reached full stability at the end. Perturbation in the compactness of the wild-type system was observed at different time intervals. For instance, the Rg value during the first 130 ns faced significant deviation, and afterward, the Rg graph was seen to flatten, but the average Rg remained high. The GRP78-P.1 variant was found to have considerable compactness and retained its 3D conformation, except for a few adjustments. The GRP78-B.1.351 variant experienced a steady increase in the plot (showing continuous small structural changes) before reaching significant stability halfway. The Rg value for GRP78-B.1.351 then decreased, and the structure remained more compact. The GRP78-B.1.617 variant received minor structural deviations but remained relatively consistent overall in terms of structural stability. During the last 100 ns, all the systems demonstrated a more compact behavior. This reveals that the binding and unbinding events that occurred during the simulation and the interruption of interfacial water caused variations in the structural compactness. The Rg(s) of all the complexes are given in Figure 6.

### 3.5. Residue Level Flexibility Analysis

**Figure 7:**
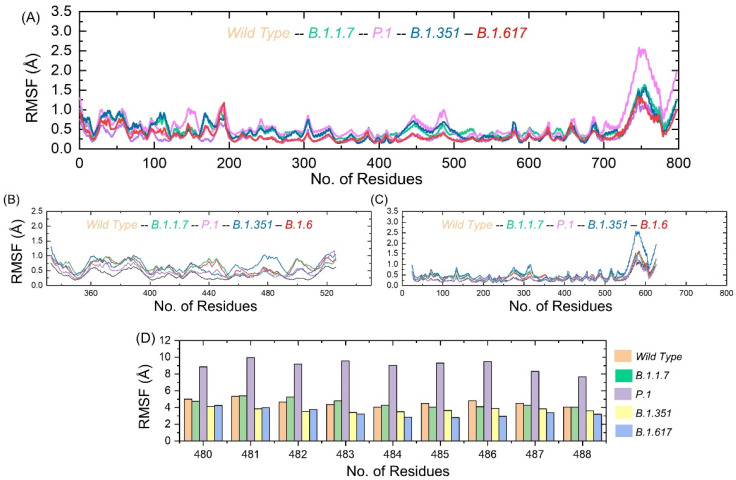
Residual flexibility of GRP78 and spike RBD of the wild-type and variants calculated as RMSF. (**A**) shows the RMSF of wild-type and variants RBD-GRP78 complex; (**B**) shows the RMSF of RBD only; (**C**) shows the RMSF of GRP78 only; (**D**) shows the RMSF of the region C480–C488.

Understanding residue level flexibility of the systems is key to highlighting residues that are vital in holding the interacting ligand and overall stabilization of the complex. As can be seen in Figure 7A, the majority of the residues of the systems are in a significant state of equilibrium, with a mean RMSF of around 1 Å. However, the residue ranges from 720–800 showed some flexibility, which is still highly acceptable in terms of stability. Figure 7B presents the RMSF of the wild-type and variant RBDs displaying different flexibilities. A higher RMSF was observed between 350 and 380 residues. The region between 460 and 520 also exhibited higher flexibility. This region is the interaction site for RBD and GRP78. Figure 7C shows the GRP78 only where the flexibility was higher in the regions of 260–320 and 540–624. In order to provide further insights into the binding site of RBD (C480–C488), we calculated the residual flexibility. The results showed that the three loops in the spike RBD domain γ1 (474–485), γ2 (488–490), and γ3 (494–505) previously reported to have higher flexibility also demonstrated similar results by enhancing the residual flexibility of between 480–488 aa, particularly in the P.1 variant [15]. Figure 7D shows that this region possesses comparable flexibility except for the P.1 variant. Thus, the difference in dynamic flexibility results in variable conformational optimization and binding with GRP78.

### 3.6. Analysis of Intermolecular Hydrogen Bonding

**Figure 8:**
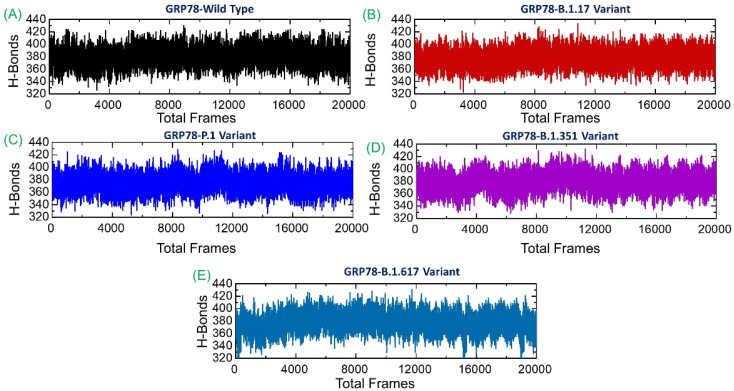
Hydrogen bonding analysis of the wild-type and variant’s complexes. (**A**) shows the total H-bonds of wild-type-RBD-GRP78 complex; (**B**) shows the total H-bonds of B.1.1.7-RBD-GRP78 complex; (**C**) shows the total H-bonds of P.1-RBD-GRP78 complex; (**D**) shows the total H-bonds of B.1.351-RBD-GRP78 complex; (**E**) shows the total H-bonds of B.1.617-RBD-GRP78 B.

Protein–protein association is mainly guided by a variety of factors, among which hydrogen bonds and hydrophobic interactions are the key players. The interaction of protein interfaces is always occupied by water molecules that compete with the hydrogen bonding between the residues. The processes behind protein–protein coupling and the extent to which hydrogen bonds play a role in this association are unknown [38]. Whether hydrogen bonds govern protein-protein docking, in particular, is a long-standing concern with poorly understood mechanisms [14,15]. Hydrogen bonding is a crucial stabilizing factor in the formation of biological complexes. These bonds are formed when hydrogen is shared between highly electronegative atoms. In the wild-type, the average number of hydrogen bonds during the simulation was reported to be 384; for B.1.1.7, the average hydrogen bonds were 392; in P.1 variant, 386; in B.1.351, 389; and in B.1.617, the average number of hydrogen bonds was 390. All the studied systems revealed a high number of hydrogen bonds, which are subjected to continuous formation/breaking in the entire simulation time. This demonstrates that the interactions between GRP78 and variants are enriched with strong hydrogen bonding and show excellent binding affinity compared to the wild-type. Interestingly, the three hydrogen bonds established by Lys484 (also by specific substitution at this position) are reported to be strongly preserved here and thus corroborate the previous findings [12]. The total number of hydrogen bonds in each system is shown in Figure 8.

### 3.7. Estimation of Binding Free Energy

The strength of a biomolecular association can be determined by estimating the binding affinity of the two interacting macromolecules. Computations of binding free energy using MM/GBSA methods are the most commonly used approach to re-rank docking conformations via calculations of structural-dynamic stability, the strength of interacting key hotspots, and total binding energies. The aforesaid method is computationally less expensive than any other method, i.e., the alchemical free energy calculation method. The MM/GBSA technique is considered to be more accurate and comprehensive than the conventional scoring functions. Thus, to re-evaluate the binding scores of the wild-type and variant complexes, we employed the MM/GBSA approach using 20,000 structural frames (Table 2). In the case of the wild-type, the vdW energy was reported to be −76.54 kcal/mol; for B.1.1.7 variant (−68.88 kcal/mol); for P.1 variant (−84.05 kcal/mol); for B.1.351 (−72.32 kcal/mol); while for B.1.617 (−68.15 kcal/mol). The electrostatic interactions for the complexes were reported to be −453.55 kcal/mol (wild-type); −180.57 kcal/mol (B.1.1.7); −280.96 kcal/mol (P.1); −436.79 kcal/mol (B.1.351); and for B.1.617, the electrostatic energy was reported to be −560.29 kcal/mol. The total binding energy for each complex was reported to be −43.45 kcal/mol (wild-type); −57.48 kcal/mol (B.1.1.7); −59.74 kcal/mol (P.1 and); −46.87 kcal/mol (B.1.351); while for B.1.617, the total binding energy was reported to be −37.69 kcal/mol. The current findings strongly corroborate with the previous findings, where higher infectivity was associated with the higher total binding energy induced by mutations in the RBD in different variants [12,15,17]. From the preceding, the three variants B.1.1.7, P.1, and B.1.351 exhibit stronger affinity toward GRP78 and thus may increase their infectivity more robustly than others.

## 4. Conclusions

The current study provides a structural basis for the binding of GRP78 to the different variants of SARS-CoV-2 using a biomolecular simulation approach. We conclude that the binding affinity of B.1.1.7, P.1, and B.1.351 variants increases due to the bonding network variation, which might help the virus enforce a higher infectivity and disease severity. Our analysis revealed that these variants possess higher docking scores toward ACE2 than the wild-type, which is due to the increased number of hydrogen bonds and salt bridges. The dynamic structural features further established that these variants exhibit stable dynamics, which follows the global stability trend. The dynamic structural features further validated the findings and revealed that these stabilizing mutations are responsible for stronger binding and sustainability of essential hydrogen bonds. Moreover, the conclusions from the MM/GBSA results further demonstrated a higher binding affinity by these variants toward ACES. Other polymorphic sites should be explored, and essential features should be demonstrated so that the final therapeutic strategy can be mapped. In conclusion, this study provides a strong impetus for the development of novel drugs against these new variants.

## Figures and Tables

**Table 1:** Bonding pattern, docking scores, and dissociation constant of each complex are outlined.

Complexes	Wild-Type	B.1.1.7 Variant	P.1 Variant	B.1.351 Variant	B.1.617 Variant
Interface residues	13,13	14,16	12,11	12,12	12,13
Salt bridges	***	1	2	***	***
Disulfide bonds	***	***	***	***	***
Hydrogen bonds	4	7	7	7	4
Non-bonded contact	67	75	50	83	70
Docking scores	−205.36	−289.67	−279.59	−265.66	−224.32
Dissociation constant (K_D_)	4.02 × 10^−8^	3.00 × 10^−4^	3.00 × 10^−7^	3.65 × 10^−7^	4.01 × 10^−5^

(***) means no detected.

**Table 2:** MM/GBSA binding free energies of the wild-type and all the variants. All energy values are presented in kcal/mol.

	Wild-Type	B.1.1.7 Variant	P.1 Variant	B.1.351 Variant	B.1.617 Variant
Van der Waals	−76.54	−68.88	−84.05	−72.32	−68.15
Electrostatic Interactions	−453.55	−180.57	−280.96	−436.79	−560.29
Generalized Born	495.75	201.03	315.11	472.59	601.11
Non-polar Solvation Energy	−9.11	−9.06	−9.84	−10.35	−10.36
Total Binding Energy	−43.45	−57.48	−59.74	−46.87	−37.69

## Data Availability

All the data is available on RCSB, UniProt, and any simulation data would be provided on reasonable demand. The accession numbers to access this data are given in the manuscript.
